# Our Book Table

**Published:** 1885-06

**Authors:** 


### OUR BOOK TABLE.

Modern Therapeutics of the Diseases of Children, with Observations on the Hygiene of Infancy; by Joseph F. Edwards, M. D. Editor of the Annals of Hygiene; Associate editor of the Medical and Surgical Reporter; author of "Bright's Disease and its Treatment,"; Fellow of the College of Physicians of Philadelphia, etc., etc. Philadelphia: D. G. Brinton, 1885.

When a book is announced as having been written and prepared in the office of *The Medical and Surgical Reporter*, it may safely be assumed that it is well worth attention. Dr. Brinton and his staff have too much of knowledge and experience to permit serious mistakes in their estimate of medical literature. So, when we are told that this work is prepared by one of the editors of *The Reporter*, and that it is published by the editor in chief of that sterling journal, little more need be said.

The plan of the work differs materially from that of other modern works on the ailments of children. In the first place, it is devoted mainly to the therapeutics of disease. Then again, instead of being a continued series of chapters by one author, each section devoted to some particular phase of disease, the method of treatment of the most eminent foreign and American specialists is presented under each head. The sum of the wisdom and experience of all those most competent to advise is thus epitomized, and instead of the opinion of one man, there is given the judgment of many. In addition, the latest writings and most recent discoveries are summarized, and the whole given in a convenient form. Thus the results of the experience of ages and the last new investigations, are presented side by side. Under the head of "Diarrhœal Disorders," for instance, the experience, methods of treatment, and usual prescriptions of no less than twenty-seven eminent practitioners of America and Europe are given, besides a syllabus of the most important articles from different publications, and a general table of "Notes on Remedies." When it is remembered that all this great mass of information is completely indexed, so that it is easy to turn at once to the opinion of any authority on any subject, or to notes on any special remedy, or to particular phases of any disease, it may readily be seen what an invaluable work of reference upon the therapeutics of an important class of diseases is here presented.

